# Moderate Multiple Parentage and Low Genetic Variation Reduces the Potential for Genetic Incompatibility Avoidance Despite High Risk of Inbreeding

**DOI:** 10.1371/journal.pone.0029636

**Published:** 2012-01-03

**Authors:** Cristina Tuni, Sara Goodacre, Jesper Bechsgaard, Trine Bilde

**Affiliations:** 1 Department of Bioscience, Aarhus University, Aarhus, Denmark; 2 Institute of Genetics and School of Biology, University of Nottingham, Nottingham, United Kingdom; Macquarie University, Australia

## Abstract

**Background:**

Polyandry is widespread throughout the animal kingdom. In the absence of direct benefits of mating with different males, the underlying basis for polyandry is enigmatic because it can carry considerable costs such as elevated exposure to sexual diseases, physical injury or other direct fitness costs. Such costs may be balanced by indirect genetic benefits to the offspring of polyandrous females. We investigated polyandry and patterns of parentage in the spider *Stegodyphus lineatus*. This species experiences relatively high levels of inbreeding as a result of its spatial population structure, philopatry and limited male mating dispersal. Polyandry may provide an opportunity for post mating inbreeding avoidance that reduces the risk of genetic incompatibilities arising from incestuous matings. However, multiple mating carries direct fitness costs to females suggesting that genetic benefits must be substantial to counter direct costs.

**Methodology/Principal Findings:**

Genetic parentage analyses in two populations from Israel and a Greek island, showed mixed-brood parentage in approximately 50% of the broods. The number of fathers ranged from 1–2 indicating low levels of multiple parentage and there was no evidence for paternity bias in mixed-broods from both populations. Microsatellite loci variation suggested limited genetic variation within populations, especially in the Greek island population. Relatedness estimates among females in the maternal generation and potentially interacting individuals were substantial indicating full-sib and half-sib relationships.

**Conclusions/Significance:**

Three lines of evidence indicate limited potential to obtain substantial genetic benefits in the form of reduced inbreeding. The relatively low frequency of multiple parentage together with low genetic variation among potential mates and the elevated risk of mating among related individuals as corroborated by our genetic data suggest that there are limited actual outbreeding opportunities for polyandrous females. Polyandry in *S. lineatus* is thus unlikely to be maintained through adaptive female choice.

## Introduction

Polyandry - female mating with multiple males - may carry considerable costs of mating from elevated exposure to predation, sexual diseases or physical injury [1], [2], [3]. Despite the costs, polyandrous mating systems are widespread and occur in most animal taxa [4]. Polyandrous females may achieve direct (material) fecundity enhancing benefits from resources provided by the male such as nutrient donations (e.g. nuptial gifts or nutritious ejaculates) or parental care [5], [6]. Polyandrous females may also derive indirect (genetic) benefits in the form of additive (i.e. good genes) or non-additive (i.e. genetic compatibility) genetic effects that enhance offspring viability [7], [8], [9], [10], [11]. Genetic benefits are expressed through improvement of fitness traits such as egg hatching rate, survival, resistance against diseases, or the reproductive success of the offspring [12], [13], [14]. Genetic benefits of polyandry in the form of increased genetic compatibility may require post-copulatory fertilization bias through cryptic gamete choice or sperm competition [15], [16]. Empirical evidence shows that females with the opportunity for post-copulatory sexual selection can bias fertilization towards sperm of unrelated males, suggesting that polyandry is a mechanism to avoid the genetic incompatibilities that arise from inbreeding [17], [18], [19], [20], [21]. Balancing the costs of mating with direct and indirect benefits of mate choice should shape female optimal mating rate. Alternatively, differential selection among the sexes on optimal mating rate may lead to suboptimal mating patterns for females, if sexual conflict and sexually antagonistic co-evolution lead females to engage in superfluous matings [3], [22], [23], [24].

Natural mating rates are poorly known in invertebrates and polyandrous mating systems are mostly studied in laboratory settings. Moreover, the number of males that a female mates with does not necessarily represent the number of sires that gain a share in paternity, as post-copulatory sexual selection may cause fertilization biases and differential patterns of parentage within a brood. Data on patterns of parentage in natural populations may provide insights to the potential for sperm competition and sperm selection as forces underlying the evolution of polyandry [15].

In this study, we performed genetic parentage analysis in two natural populations of the spider *Stegodyphus lineatus* (Araneae: Eresidae) to investigate whether polyandry and mixed paternity is part of the reproductive biology of this species, and to assess the potential for polyandrous females to acquire genetic benefits in the form of reduced risk of inbreeding. *Stegodyphus lineatus* are philopatric, which results in natural aggregations of related individuals [25], [26]. Individuals commonly mate within natal patches, which increases the risk of inbreeding as there is no apparent pre-copulatory discrimination against relatives as mates [25]. Females are sedentary and may be visited by 1–5 males during the mating season [27], [28], [29]. However, it is not known whether or not these males actually copulate and hence the degree of polyandry and mixed-brood parentage in the wild remains unknown. Under experimental conditions, females indiscriminately accept and copulate with the first male they encounter, while they are resistant towards subsequent males and may engage in physical combats to avoid re-mating [30], [31]. Sperm transfer is not associated with material benefits, and polyandry carries net costs to females from prolonged oviposition time, fewer surviving offspring, lower offspring body mass, and increased risk of brood failure [28], [31]. Males may occupy female nests and webs for several days, and females suffer direct fitness costs of male cohabitation through male kleptoparasitism which negatively affects female body condition and hence fecundity [29], [32].

For polyandry to be adaptive in the face of these substantial direct costs, the magnitude of indirect genetic benefits should be large [10], [33], [34], [35]. Under this scenario, we expect polyandrous females to acquire indirect benefits through post-mating sexual selection of un-related and/or genetically dissimilar males. In the absence of direct fitness benefits, female preference for outbred males could evolve and be maintained in small populations where parent-offspring correlations in heterozygosity or inbreeding may arise providing outbreeding benefits [36]. Alternatively, polyandry may be non-adaptive, for example as a result from male coercion that results in sub-optimal mating rates for females [3]. We determined patterns of parentage in two populations of *S. lineatus* (from Israel and Greece) by genotyping wild-caught females and their broods using microsatellite markers [37]. We assessed the potential for polyandrous females to acquire indirect genetic benefits for their offspring in the form of inbreeding avoidance by i) estimating genetic diversity within populations; ii) estimating relatedness among potentially interacting individuals, and iii) testing whether there is paternity bias within broods that could indicate potential post-copulatory processes.

## Methods

### Study Organism

*Stegodyphus lineatus* (Latreille 1817) is a web-building spider with an annual life cycle occupying arid and semiarid habitats around the Mediterranean basin. Individuals occupy silk retreats built on shrubs, which are abandoned by adult males that start wandering in search of sedentary females. The mating season starts in March and eggs are laid from April to June [27], [29]. Females provide maternal care by tending the eggs, releasing the young and feeding them by regurgitation. Approximately two weeks after hatching the offspring consume the mother and show philopatric dispersal out of the natal nest 2–4 weeks after matriphagy.

### Sampling, DNA extraction and Genotyping

We collected spiderlings at a pre-dispersal stage from their natal nest together with (where possible) remnants of the eaten mother (i.e. the exoskeleton). Sampling occurred from two geographically separated populations during summer 2006, from the Negev Desert (Israel) and at the island of Karpathos (Greece). Spiders are not evenly distributed within habitats, they are found in patches or sites where a number of individuals are in close proximity of each other reflecting philopatric dispersal [26], [38]. Spiders occurring within a defined geographical area with more or less continuous suitable habitat were regarded as one population, whereas collection sites within the population were distinct patches with spiders of a distance of at least 3 km to the next site. We considered this a useful scale to implement, since population genetic studies indicate genetic differentiation at this level (Bilde et al 2005).

We collected a total of 10 broods with a mean number of offspring per brood (±SE) of 46.8 (±8.6) from 2 sites in the Negev Desert; 7 broods from Sede Boker (SB-) and 3 from Lehavim (L-) (mothers' exoskeleton collected from a total of 8 nests). We collected a total of 40 broods with a mean number of offspring of 25.17 (±2.5) from 4 sites in Karpathos; 12 broods in site 1 (S1-), 15 in site 2 (S2-), 7 in site 3 (S3-) and 6 in site 4 (S4-) (mother's exoskeleton collected from a total of 29 nests). All samples were preserved in 96% ethanol. DNA was extracted using a Chelex protocol [39] and stored at −20°C. Tissue of approximately 2 mm^3^ of size (from offspring body or female exoskeleton) was dried at 60°C for 30 minutes, homogenized and 200 µl of 20% Chelex ® resin solution (Bio-Rad) was added. Samples were vortexed briefly, boiled at 100°C for 10–12 minutes in a heating block and centrifuged for 3 minutes at 13000 rpm. Samples were genotyped using five nuclear polymorphic microsatellite loci [37] by NGB Genetics (Bologna, Italy) using fluorescent-labeled primers (ABI™) and fragments were sized on an ABI sequencer 3700 machine.

The study did not require any specific permit and did not involve endangered or protected species.

### Data Analysis

Prior to analyses, genotypes of offspring were screened and loci that showed absence of a maternal allele from the known mother were removed from the analysis. Presence and frequency of null alleles was tested for each locus using the software MICRO-CHECKER [40] that estimates null alleles based on the excess of homozygotes when evenly distributed across the homozygote classes of alleles. Finally, since the power to detect multiple paternity depends on the level of marker polymorphism [41] we assessed the statistical power of genetic analysis to detect mixed paternity broods using the software PrDM [42]. PrDM accounts for the effects of population allele frequencies, number of loci, number of alleles, clutch size, number of putative fathers and their respective reproductive success.

We adopted three different methods to obtain estimates of parentage within broods and to compare estimates among methods, allele count, GERUD 2.0 [43] and PARENTAGE 1.0 [44], as there is no consensus in the literature on the use of a specific method. Allele count involved identifying and discounting the maternal allele from each offspring at each locus separately by comparing the progeny genotypes to that of their mother. The remaining alleles were inferred as paternal alleles and multiple paternity was confirmed when more than two paternal alleles were found at a single locus. An estimate of the number of fathers present was calculated dividing the number of paternal alleles by 2, since each potential father can donate two different alleles [45], [46]. Broods with missing maternal genotype (2 from Israel and 11 from Greece) were excluded from the analysis. Allele count is a conservative method which takes into account each locus separately. Unlike allele counting, the software GERUD uses multiple loci simultaneously for inferring the number of sires. Maternal alleles are removed from offspring genotypes, paternal alleles are derived and all possible paternal genotypes simulated before calculating the combination that yields the fewest number of contributing sires. GERUD can reconstruct parental genotypes when maternal genotypes are missing. However, the software does not take into account population allele frequencies and provides the minimum number of fathers rather than a probable number. In addition, individuals with missing genotypes at one or more loci were removed from the analyses due to the inability of the software for processing missing alleles. Finally, PARENTAGE 1.0 is the less conservative methods that uses Bayesian statistics and takes population allele frequencies into account and prior information of what is known about the parentage of the offspring, such as maternal genotypes and number of fathers. Allele frequencies were estimated based on background maternal allele frequencies from the target sample site only. Based on the observed number of visiting males in a females nest [29], [47] an estimate of expected number of sires was approximated by a gamma distribution (gamma(2,1)) and used as prior information in the parentage analysis. We ran 5000 iterations for each family (burn-in 200,000; thinning interval 400). In addition, for each iteration PARENTAGE estimates the proportional parentage of each father to the offspring within a brood. To assess evidence for paternity skew we tested whether the relative proportion of parentage from each sire within a brood differed from an even contribution from each sire. For each iteration, the relative paternity share for each possible sire was estimated as the sum of squared proportion of offspring assigned to each father [46]. This was done for each number of fathers in the estimated range for each brood, and for each estimated number of sires (number of sires >1) we tested whether the relative parentage differed from an equal contribution. Simulations were done using the actual number of offspring in the given nest and assuming equal probability for each father to sire the offspring. Each father was assigned 1 offspring before simulations to avoid fathers siring no offspring.

Levels of genetic variation of sires and inbreeding were calculated within each population using GENEPOP 3.3 [48] measuring the number of alleles, observed (H_o_), expected (H_e_) heterozygosity and deviations from Hardy-Weinberg equilibrium. We applied FSTAT [49] for calculation of allelic richness and estimates of *F*is, the inbreeding coefficient. Calculations were performed by random sampling of one offspring from each nest. Relatedness estimates among the parental and offspring generation within each site of collection were obtained by determining the mean of all pair wise relatedness values among adult females and among offspring of different broods (one offspring randomly sampled by each nest) separately by using the software ML-RELATE [50]. We note that the loci used in this study were those that were most polymorphic among several candidate loci suggesting that we are not underestimating genetic variation, but rather confirm general low levels of genetic variation in this species (Jesper Bechsgaard, unpublished data).

## Results

All five microsatellite loci showed low variability (range of scored number of alleles 1–5). Overall, observed heterozygosity varied considerably among loci (from 0 to 0.65). No linkage disequilibrium was detected between any loci (P>0.05) in any population. Population genetic variables are showed in Table 1. The Greek population was monomorphic at two loci (R7 and R7N). Significant departure from Hardy-Weinberg genotypic proportions were found in the Israeli population (χ^2^ = 25.52; d.f. = 10; P = 0.0046) due to heterozygote deficiency for locus R5N (Test for H deficit: P = 0.0067; S. E. = 0.0008) and in the Greek population (χ^2^ = 23.39; d.f. = 6; P = 0.0007) due to heterozygote deficiency for loci R4 and R5N (Test for H deficit R4: P = 0.001; S.E. = 0.0004; R5N: P = 0.0038; S. E. = 0.008). *F*is inbreeding coefficient values did not differ significantly among populations and were significantly greater than zero when estimated over all loci (Israel, *F*is ±S.E. = 0.44±0.1; 95% C. I. = 0.27–0.62; Greece, *F*is ±S.E. = 0.3±0.03; 95% C. I. = 0.21–0.33) indicating non-random mating within the population (Table 1).

**Table 1:** Summary statistics for 5 microsatellite loci including number of individuals analyzed (N), number of alleles (NA), expected (He) and observed (Ho) heterozygosity, allelic richness, estimates of inbreeding coefficient (F*is*), and relatedness (R) among offspring and adult females and percentage of full- sib (FS) and half-sib (HS) relationships between pairs of individuals.

		Offspring generation								Parental generation			
	Site	N	NA (range)	Ho/He	Allelic Richness	F*is*	R (95% C .I.)	%FS	%HS	N	R (95% C .I.)	%FS	%HS
Israel	SB	7	1–4	0.32/0.61*	2.6	0.48	0.17 (0.08;0.25)	14	24	5	0.11 (0.03;0.17)		20
	L	3	1–5	-	-	-	0.38 (0.31;0.44)	67	33	3			
Greece	S1	12	1–3	0.26/0.28	1.65	0.08	0.22 (0.15;0.28)	37	63	10	0.28 (0.17;0.39)	35	13
	S2	15	1–3	0.15/0.26*	1.58	0.46	0.25 (0.19;0.30)	42	4	9	0.26 (0.20;0.32)	38	4
	S3	7	1–3	0.26/0.32	1.79	0.17	0.19 (0.09;0.29)	28.5		6	0.22 (0.03;0.4)	27	
	S4	6	1–2	0.23/0.26	1.65	0.46	0.22 (0.02;0.43)	20	13	4	0.38 (0.09;0.66)	30	10

*denotes a significant deviation from Hardy-Weinberg equilibrium (P<0.05).

**Figure 1:**
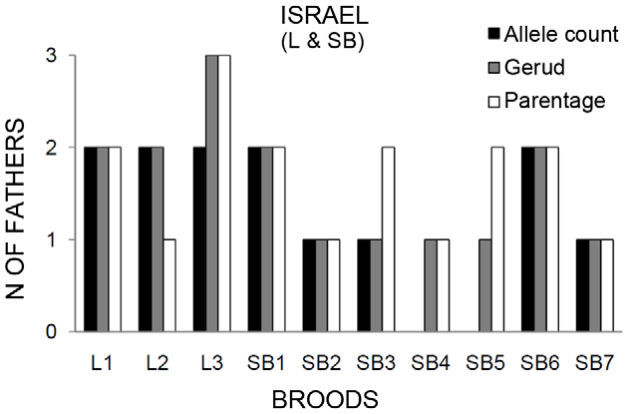
Multiple parentage in broods from the Israel population. Summary of results for number of fathers estimated by allele count, GERUD 2.0 and PARENTAGE 1.0 for the Israeli population (broods from site L- and Site SB-).

**Figure 2:**
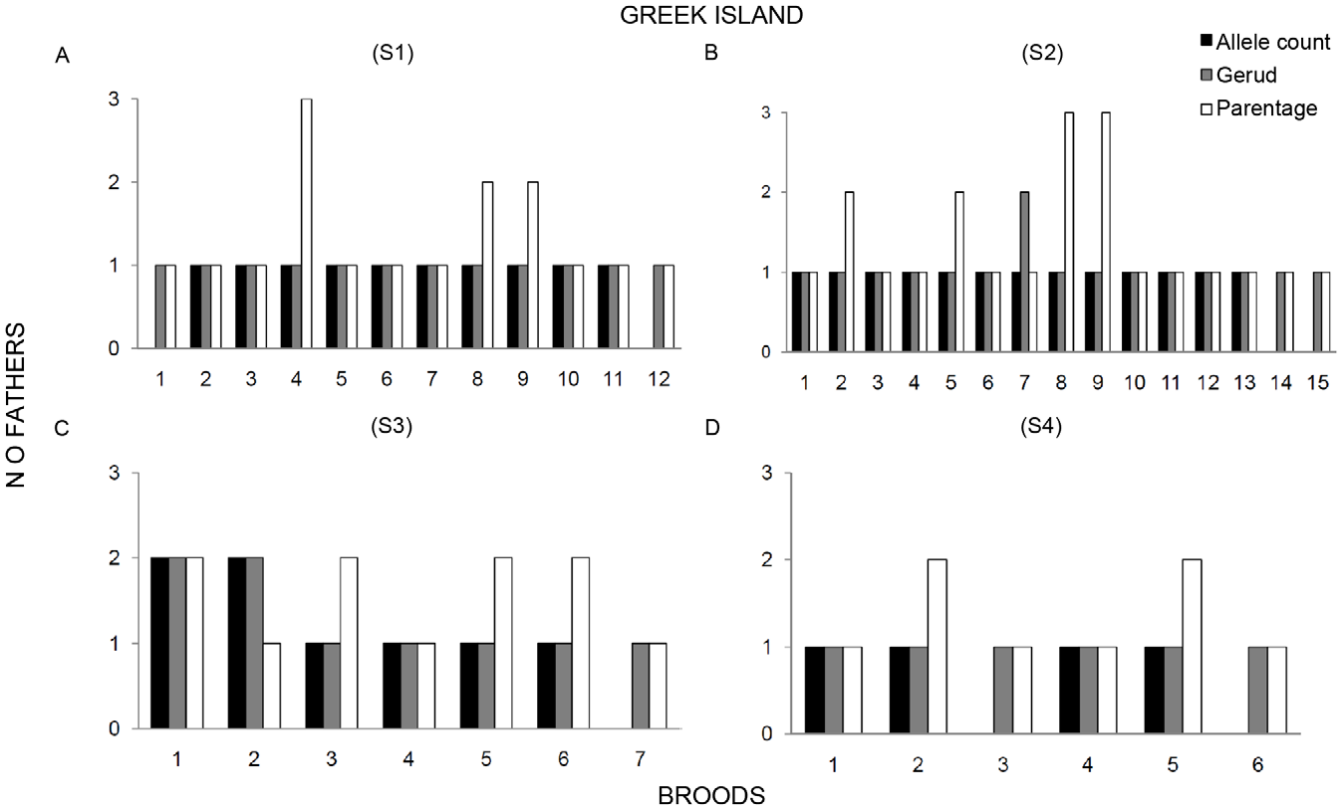
Multiple parentage in broods from the Greek island population. Summary of results for number of fathers estimated by allele count, GERUD 2.0 and PARENTAGE 1.0 for the Greek population: **a**) broods from Site 1); **b**) broods from Site 2; **c**) broods from Site 3; and **d**) broods from Site 4.

The summary of results for number of fathers estimated with allele count, GERUD 2.0 and PARENTAGE 1.0 are shown in Figures 1 & 2. Allele count revealed multiple paternity in 5 of the 8 analyzed broods from the Israeli population (Table 2) and in 2 of the 33 analyzed broods from the Greek population (Table 2), yielding a mean (±standard error) of 1.63±0.18 and 1.06±0.04 sires per brood, respectively. GERUD 2.0 estimated multiple paternity in 5 of the 10 broods from the Israeli (Table 2) and in 3 of the 40 broods from the Greek population (Table 2), with a mean of 1.6±0.16 and 1.08±0.04 sires per brood, respectively. Allele count did not reveal multiple paternity in clutch S2–7 due to missing types from the mother at one locus (Table 2). Analyses with PARENTAGE 1.0 revealed higher numbers of mixed-paternity broods. Six of the 10 broods from the Israeli population and 17 of the 40 broods from the Greek population (Table 2) showed mixed paternity broods with more than 50% probability (indicated by → in Table 2). The mean number of fathers were 1.83 for Israel and 1.73 for Greece. Skew analyses indicated skew in only one brood from each population (SB-1 and S1–4, indicated by * in Table 2). For more details about skew analyses, see Table S1.

**Table 2:** Summary of results from different methods (allele count; GERUD 2.0; PARENTAGE 1.0) for estimating the number of sires and paternity skew for *S. lineatus* wild-caught clutches from the Israeli and the Greek population.

			ALLELE COUNT			GERUD 2.0				PARENTAGE 1.0		
		Nest (# offspring)	# loci used	# paternal alleles	Min. # fathers	# offspring	# loci used	Min. # fathers	Father solutions	Mean # fathers	Proportion of iterations	Mode(p)
Israel Sites	L	1(13)	2	3	2	13	3	2	6	2.45	→0.01	2 (0.66)
		2(14)	1	3	2	14	3	2	17	1.10	0.90	1 (0.90)
		3(14)	1	4	2	7	2	3	8	3.06	→0.07	3 (0.48)
	SB	1(14)	2	3	2	10	3	2	13	2.02	→0*	2 (0.98)
		2(11)	5	2	1	11	3	1	1	1.00	1	1 (1)
		3(17)	3	2	1	18	3	1	1	1.78	→0.28	2 (0.67)
		4(41)	-	-	-	37	4	1	24	1.55	0.68	1 (0.68)
		5(64)	-	-	-	49	4	1	4	2.32	→0	2 (0.69)
		6(29)	1	3	2	26	3	2	11	2.02	→0.07	2 (0.84)
		7(33)	2	2	1	13	2	1	6	1.00	1	1 (1)
Greek Island Sites	S1	1 (14)	-	-	-	14	2	1	8	1.21	0.82	1 (0.82)
		2(34)	2	2	1	34	2	1	1	1.17	0.84	1 (0.84)
		3(12)	2	2	1	12	3	1	1	1.00	1	1 (1)
		4(60)	2	2	1	60	3	1	1	3.49	→0*	3 (0.52)
		5(15)	3	2	1	16	3	1	1	1.27	0.80	1 (0.80)
		6(83)	1	2	1	83	2	1	1	1.05	0.95	1 (0.95)
		7(34)	2	2	1	34	2	1	1	1.12	0.89	1 (0.89)
		8(23)	3	2	1	23	3	1	1	2.06	→0.22	2 (0.56)
		9(9)	3	2	1	9	3	1	1	2.13	→0.29	2 (0.44)
		10(34)	1	2	1	34	2	1	1	1.07	0.93	1 (0.93)
		11(10)	1	2	1	10	2	1	4	1.13	0.89	1 (0.89)
		12(7)	-	-	-	7	3	1	8	2.13	→0.39	1 (0.39)
	S2	1(11)	3	2	1	9	3	1	1	1.21	0.85	1 (0.85)
		2(28)	3	2	1	28	3	1	1	2.55	→0.07	2 (0.55)
		3(20)	2	2	1	20	2	1	1	2.56	→0.27	1 (0.27)
		4(6)	2	2	1	6	2	1	1	1.14	0.89	1 (0.89)
		5(9)	3	2	1	6	3	1	1	2.64	→0.19	2 (0.36)
		6(28)	3	2	1	22	3	1	1	1.63	0.64	1 (0.64)
		7(8)	2	2	1	14	3	2	17	1.34	0.76	1 (0.76)
		8(15)	1	2	1	15	2	1	1	1.53	→0.50	1(0.50)
		9(12)	4	2	1	12	3	1	1	1.02	0.97	1(0.97)
		10(28)	2	2	1	18	2	1	1	1.15	0.88	1 (0.88)
		11(8)	3	2	1	5	3	1	1	2.29	→0.34	1 (0.34)
		12(35)	2	2	1	25	2	1	1	2.90	→0.15	3 (0.38)
		13(23)	2	2	1	14	3	1	1	2.77	→0.19	3 (0.28)
		14(27)	-	-	-	16	2	1	2	1.23	0.82	1 (0.82)
		15(9)	-	-	-	9	2	1	4	2.12	→0.37	1 (0.37)
	S3	1(36)	3	3	2	19	3	2	24	1.86	→0.39	2 (0.42)
		2(22)	3	3	2	18	3	2	15	1.54	0.52	1 (0.52)
		3(22)	2	2	1	20	2	1	2	2.43	→0.15	2 (0.46)
		4(25)	3	2	1	16	3	1	1	1.20	0.86	1 (0.86)
		5(27)	3	2	1	8	3	1	1	2.36	→0.06	2 (0.62)
		6(19)	2	2	1	17	2	1	1	2.42	→0.11	2 (0.51)
		7(19)	-	-	-	19	3	1	2	1.06	0.94	1 (0.94)
	S4	1(14)	2	2	1	14	2	1	1	1.16	0.86	1 (0.86)
		2(39)	1	2	1	39	3	1	4	1.75	→0.29	2 (0.67)
		3(15)	-	-	-	15	2	1	2	1.15	0.86	1 (0.86)
		4(4)	2	2	1	5	3	1	1	1.61	0.60	1 (0.60)
		5(27)	2	2	1	25	2	1	1	2.21	→0	2 (0.81)
		6(4)	-	-	-	4	3	1	8	1.49	0.67	1 (0.67)

*→denotes that the probability of mixed paternity is higher than 50% based on estimates;*

*denotes that there is indication of skewed proportions of paternity among males siring offspring in the clutch.

Relatedness values among adult females and among offspring of different broods are shown in Table 1. ML-RELATE can adjust relatedness calculations to accommodate null alleles by using maximum likelihood estimates of the frequency of null alleles in all calculations [50]. Relatedness calculations were adjusted for the presence of null alleles in locus R5N. No significant difference in the mean relatedness estimates among the two populations was detected for adult females (test for significance t = 2.29; d. f. =  3; P = 0.94), and for the offspring generation (t = 0.66; d. f. = 4; P = 0.5). Within each site the mean genetic relatedness was greater than zero (Table 1), indicating that the most closely related individuals are also likely to be located near to one another. Relationships among individuals - full sibling (R = 0.5) and half sibling (R = 0.25) - were assessed using ML-RELATE by estimating pedigree relationships of pairs of individuals at each site separately. Among females full sibling relationships were detected exclusively in the Greek population while half-sibling relationships were revealed among adult females from both populations. Full sibling and half sibling relationships were detected among offspring of both populations (Table 1).

We note that while the model system used here is particularly useful for studying indirect selection for genetically dissimilar males, it also raises challenges in terms of low marker polymorphism due to a history of inbreeding in natural populations [25], [41]. The five microsatellite loci proved to be moderately variable, therefore we performed simulations using PrDM to assess the actual accuracy of the estimated frequency of multiple sired broods. PrDM accounts for the possibility that less than 3 alleles were detected in multiple sired broods, which may occur when 2 sires share a common genotype. Given the population allele frequencies, the average number of offspring sampled for each female (47 in Israel and 25 in Greece) and 2 sires with equal fertilization success, the probability of detecting multiple sires revealed by the software PrDM was 0.8 and 0.2 for detecting 2 fathers in the Israel and the Greek population, respectively. Thus, the probability of detecting multiple fathered broods in the Greek population is considerably lower than in Israel.

## Discussion

Our data show evidence for polyandry and mixed paternity broods in two natural populations of the spider *Stegodyphus lineatus*. We estimated parentage using three different methods and found differences among the methods. Allele count and GERUD, produced the lowest estimates of multiple parentage. These are conservative methods that may lead to an underestimation of number of fathers when different males may carry the same allele. This is likely to be the case for this species because of low population genetic variation [37]. Underestimations are more likely if males are related to each other, or if males are related to the mother, both of which could be true in our study case. PARENTAGE, by taking into account population allele frequencies, is less conservative and produced the highest estimated number of fathers [45]. The estimated number of sires was remarkably similar in both populations, (1.8 and 1.7), and notably similar to the average number of males appearing on female webs (1.5–2.1) during the mating season in a Greek [29] and a Negev population [28], respectively. Male-female cohabitation always results in mating [30], hence observed encounter rate is a reliable proxy for female mating rate. The remarkable overlap documented here between observed mating rate in the field [28], [29] and genetic representation in the brood (this study) suggests that males that acquire access to a females' web not only ensure copulation, but also very likely a share of parentage of the brood.

The molecular genetic estimates of multiple paternity may be conservative since genetic variation within populations was low, especially in the Greek island population, which is consistent with previous findings [37]. Low genetic variation likely results from philopatry, high relatedness among neighbouring spiders, and constraints on dispersal, which are factors that in concert result in a history of inbreeding in *S. lineatus* populations [25], [26]. Consistent with this scenario, our data revealed high levels of relatedness among adult females within sites. Full sibling relationships were found among females of the Greek island population (27–38% of all pairs of females within each site) while females from the Negev population were estimated to be as closely related as half-sibs (20% of all pairs within sites). Similarly, relatedness among potential interacting individuals was found to be high; 20–42% of the relationships among individuals within sites the Greek population and 14–67% of those within sites of the Israel population were estimated full sibling relationships. Because of high genetic similarities among individuals within sites, males are likely to share alleles, and hence the degree of polyandry will likely be underestimated: related males may be contributing similar genotypes to offspring within a brood, particularly if males are related among each other as predicted by natal philopatry [25], [26].

Given the direct costs of re-mating documented in this species (see references above), adaptations such as post-copulatory fertilization biases would allow polyandrous females to ameliorate these costs through indirect genetic benefits to their offspring [14], [15], [18]. Post-copulatory sexual selection may provide genetic benefits by reducing costs of inbreeding and genetic incompatibilities that result from the interaction between maternal and paternal haplotypes [12], [18], [19], [21]. Reducing the costs of inbreeding or increasing the chance of fertilizing eggs with the genetically most compatible males may be particularly important in differentiated populations with small numbers of breeding individuals since these populations are subject to loss of genetic variation [51], [52]. Indeed, *S. lineatus* populations experience inbreeding which may depress fitness [53], [54]. Asymmetrical paternity distributions in the brood may indicate potential cryptic processes of post mating sexual selection. We found very little evidence for paternity skew in broods from both populations. However, we do not have the power to reject the hypothesis for post mating selection of unrelated males. In a previous study, lack of apparent female pre-copulatory inbreeding avoidance mechanisms, together with a very moderate fitness decline in experimental matings among sib mates suggest that costs of inbreeding in this species are relatively low [25]. Low costs of inbreeding, low genetic variation among potential mates and an elevated risk of mating among related individuals as corroborated by our genetic data may suggest that there are limited actual outbreeding opportunities for polyandrous females.

For the reasons outlined above, we are unable to detect much potential for polyandrous females to acquire indirect genetic benefits in the form of reduced inbreeding. Notably, the strength of indirect selection on net fitness must be substantial to balance direct costs of polyandry. Even if genetic benefits are present but difficult to detect, we propose that they are unlikely to counter high costs of polyandry in our study system: Firstly, indirect selection on female preferences via indirect genetic effects is likely to be weak in relation to direct selection [33], [34], [55]. For example, offspring are related to male parents who might possess favored traits by ½, and they are related to their female parent who prefers such males also by ½. These combined probabilities reduce the intensity of selection on female preference traits to less than 1/4 of what they might be if selection were acting on female preference traits directly [35]. The more males that hold a share of paternity within a clutch, the lower the fraction of offspring carrying the preferred allele, which further weakens the strength of indirect selection. Secondly, the evolution of adaptive female choice (pre or post-copulatory) relies on adequate presence of genetic variation for fitness: for genetic benefits to exert indirect selection on female mating preferences the variation in genetic quality of potential mates must be very large to bear the direct costs [10], [56], [57]. In *S. lineatus*, it is possible that adequate genetic variation is not present, as genetic variation was low (this study; [26]), and males mate within their natal patch which increases the likelihood of mating with a relative [25]. Polyandrous females are thus unlikely to experience sufficient genetic variability among their mates to acquire substantial indirect benefits of mate choice.

We here present one of the first reports in spiders using co-dominant markers to assess natural rates of polyandry and patterns of parentage [58]. Previous studies have revealed mixed paternity in natural populations in a broad range of taxa including fish, birds and insects [45], [59], [60], [61].

We show that genetic variation within two natural populations is low, and that relatedness among potential mates is high (R approximately 0.2). Due to the low genetic variability of mating partners the potential for polyandrous females to gain indirect genetic benefits through post-mating sexual selection may be low, suggesting that genetic benefits in the form of reduced inbreeding are unlikely to compensate entirely for the direct costs of re-mating. Polyandry in *S. lineatus* is thus unlikely to be maintained by adaptive female choice.

## Supporting Information

Table S1 **Skew analyses.** PARENTAGE 1.0 estimates the contribution of each father to the offspring. For each iteration we calculated paternity skew as the sum of squared proportion of offspring assigned to each father (Simmons *et al.* 2007). For each number of fathers in the estimated range we tested the average skew against a simulated distribution. Simulations were done using the actual number of offspring in the given nest and equal probability for each father to sire the offspring. Each father was assigned 1 offspring before simulations to avoid fathers siring no offspring.
(DOC)

## References

[1] Daly M (1978). The cost of mating.. The American Naturalist.

[2] Knell RJ, Webberley KM (2004). Sexually transmitted disease of insects: distribution, evolution, ecology and host behaviour.. Biological reviews of the Cambridge Philosophical Society.

[3] Arnqvist G, Rowe L (2005). Sexual Conflict.

[4] Birkhead TR, Møller AP (1998). Sperm Competition and Sexual Selection.

[5] Arnqvist G, Nilsson T (2000). The evolution of polyandry: Multiple mating and female fitness in insects.. Animal Behaviour.

[6] Thornhill R, Alcock J (1983). The Evolution of Insect Mating Systems.

[7] Yasui Y (1997). A “good sperm” model can explain the evolution of costly multiple mating by females.. The American Naturalist.

[8] Zeh JA, Zeh DW (1996). The evolution of polyandry I: intragenomic conflict and genetic incompatibility.. Proceedings of the Royal Society London B.

[9] Puurtinen M, Ketola T, Kotiaho JS (2005). Genetic compatibility and sexual selection.. Trends in Ecology & Evolution.

[10] Bilde T, Friberg U, Maklakov AA, Fry JD, Arnqvist G (2008). The genetic architecture of fitness in a seed beetle: assessing the potential for indirect genetic benefits of female choice.. BMC Evolutionary Biology.

[11] García-González F, Simmons LW (2005). The evolution of polyandry: intrinsic sire effects contribute to embryo viability.. Journal of Evolutionary Biology.

[12] Newcomer SD, Zeh JA, Zeh DW (1999). Genetic benefits enhance the reproductive success of polyandrous females.. Proceedings of the National Academy of Sciences of the United States of America.

[13] Foerster K, Delhey K, Johnsenm A, Lifjeld JT, Kempenaers B (2003). Females increase offspring heterozygosity and fitness through extra-pair matings.. Nature.

[14] Jennions MD, Petrie M (2000). Why do females mate multiply? A review of the genetic benefits.. Biological Reviews of the Cambridge Philosophical Society.

[15] Simmons LW (2005). The evolution of polyandry: sperm competition, sperm selection and offspring viability.. Annual Review of Ecology, Evolution and Systematics.

[16] Tregenza T, Wedell N (2000). Genetic compatibility, mate choice and patterns of parentage.. Molecular Ecology.

[17] Olsson M, Shine R, Madsen T, Gullberg A, Tegelström H (1996). Sperm selection by females.. Nature.

[18] Tregenza T, Wedell N (2002). Polyandrous females avoid costs of inbreeding.. Nature.

[19] Bretman A, Newcombe D, Tregenza T (2009). Promiscuous females avoid inbreeding by controlling sperm storage.. Molecular Ecology.

[20] Bretman A, Wedell N, Tregenza T (2004). Molecular evidence of post-copulatory inbreeding avoidance in the field cricket *Gryllus bimaculatus*.. Proceedings of the Royal Society London B.

[21] Simmons LW, Beveridge M, Wedell N, Tregenza T (2006). Postcopulatory inbreeding avoidance by female crickets only revealed by molecular markers.. Molecular Ecology.

[22] Holland B, Rice WR (1999). Experimental removal of sexual selection reverses inter-sexual antagonistic coevolution and removes a reproductive load.. Proceedings of the National Academy of Sciences of the United States of America.

[23] Rice WR (1996). Sexually antagonistic male adaptation triggered by experimental arrest of female evolution.. Nature.

[24] Zeh JA, Zeh DW (2003). Toward a new sexual selection paradigm: Polyandry, Conflict and Incompatibility.. Ethology.

[25] Bilde T, Lubin Y, Smith D, Schneider JM, Maklakov AA (2005). The transition to social inbred mating systems in spiders: role of inbreeding tolerance in a subsocial predecessor.. Evolution.

[26] Johannesen J, Lubin Y (1999). Group founding and breeding structure in the subsocial spider *Stegodyphus lineatus* (Eresidae).. Heredity.

[27] Schneider JM, Lubin Y (1997). Infanticide by males in a spider with suicidal maternal care, *Stegodyphus lineatus* (Eresidae).. Animal Behaviour.

[28] Maklakov AA, Bilde T, Lubin Y (2005). Sexual conflict in the wild: elevated mating rate reduces female lifetime reproductive success.. The American Naturalist.

[29] Schneider J (1997). Timing of maturation and the mating system of the spider, *Stegodyphus lineatus* (Eresidae): how important is body size? .. Biological Journal of the Linnean Society.

[30] Schneider JM, Lubin Y (1996). Infanticidal male eresid spiders.. Nature.

[31] Maklakov AA, Lubin Y (2004). Sexual conflict over mating in a spider: increased fecundity does not compensate for the costs of polyandry.. Evolution.

[32] Erez T, Schneider JM, Lubin Y (2005). Is male cohabitation costly for females of the spider *Stegodyphus lineatus* (Eresidae)?. Ethology.

[33] Kirkpatrick M, Barton NH (1997). The strength of indirect selection on female mating preferences.. Proceedings of the National Academy of Sciences of the United States of America.

[34] Arnqvist G, Kirkpatrick M (2005). The evolution of infidelity in social monogamous passerines: the strength of direct and indirect selection on extrapair copulation in females.. The American Naturalist.

[35] Wade MJ, Shuster SM, Demuth JP (2003). Sexual selection favors female-biased sex ratios: the balance between the opposing forces of sex-ratio selection and sexual selection.. The American Naturalist.

[36] Fromhage L, Kokko H, Reid JM (2009). Evolution of mate choice for genome-wide heterozygosity.. Evolution.

[37] Bilde T, Tuni C, Cariani A, Santini A, Tabarroni C (2009). Characterisation of microsatellite loci in the subsocial spider Stegodyphus lineatus, (Araneae: Eresidae).. Molecular Ecology Resources.

[38] Johannesen J, Lubin Y (2001). Evidence for kin-structured group founding and limited juvenile dispersal in the sub-social spider Stegodyphus lineatus (Araneae, Eresidae).. The Journal of arachnology.

[39] Villesen P, Fredsted T (2006). Fast and non-invasive PCR sexing of primates: apes, Old World monkeys, New World monkeys and Strepsirrhines.. BMC Ecology.

[40] Oosterhout CV, Hutchinson WF, Wills DPM, Shipley P (2004). MICRO-CHECKER: software for identifying and correcting genotyping errors in microsatellite data.. molecular Ecology Notes.

[41] Sefc KM, Koblmuller S (2009). Assessing Parent Numbers from Offspring Genotypes: The Importance of Marker Polymorphism.. Journal of Heredity.

[42] Neff BD, Pitcher TE (2002). Assessing the statistical power of genetic analyses to detect multiple mating in fishes.. Journal of Fish Biology.

[43] Jones AG (2005). GERUD2.0: a computer program for the reconstruction of parental genotypes from half-sib progeny arrays with known or unknown parents.. Molecular Ecology Notes.

[44] Emery AM, Wilson IJ, Craig S, Boyle PR, Noble LR (2001). Assignment of paternity groups without access to parental genotypes: multiple mating and development plasticity in squid.. Molecular Ecology.

[45] Bretman A, Tregenza T (2005). Measuring polyandry in wild populations: a case study using promiscuous crickets.. Molecular Ecology.

[46] Simmons LW, Beveridge M, Kennington WJ (2007). Polyandry in the wild: temporal changes in female mating frequency and sperm competition intensity in natural populations of the tettigoniid *Requena verticalis*.. Molecular Ecology.

[47] Schneider J, Lubin Y (1997). Does high mortality explain semelparity in the spider *Stegodyphus lineatus* (Eresidae)?. Oikos.

[48] Raymond M, Roussett F (1995). GENEPOP (version 1.2): population genetic software for exact tests and ecumenicism.. Journal of Heredity.

[49] Goudet J (1995). FSTAT (Version 1.2): A computer program to calculate F-statistics.. J Hered.

[50] Kalinowski ST, Wagner AA, Taper M (2006). ML-RELATE: a computer program for maximum likelihood estimation of relatedness and relationship.. Molecular Ecology Notes.

[51] Keller LF, Waller DM (2002). Inbreeding effects in wild populations.. Trends in Ecology & Evolution.

[52] Reed DH, Frankham R (2003). Correlation between population fitness and genetic diversity.. Conservation Biology.

[53] Charlesworth D, Charlesworth B (1987). Inbreeding depression and its evolutionary consequences.. Annual Review of Ecology and Systematics.

[54] Pusey A, Wolf M (1996). Inbreeding avoidance in animals.. Trends in Ecology & Evolution.

[55] Houle D, Kondrashov AS (2002). Coevolution of costly mate choice and condition-dependent display of good genes.. Proceedings of the Royal Society London B.

[56] Petrie M, Doums C, Møller AP (1998). The degree of extra-pair paternity increases with genetic variability.. Proceedings of the National Academy of Sciences of the United States of America.

[57] Møller AP, Alatalo RV (1999). Good-genes effects in sexual selection.. Proceedings of the Royal Society London B.

[58] Ramirez MG, Wight EC, Chirikian VA, Escobedo ES, Quezada LK (2009). Evidence for multiple paternity in broods of the green lynx spider Peucetia viridans (Araneae: Oxyopidae).. Journal of Arachnology.

[59] Griffith SC, Owens IPF, Thuman KA (2002). Extra pair paternity in birds: a review of interspecific variation and adaptive function.. Molecular Ecology.

[60] Jensen MP, Abreu-Grobois FA, Frydenberg J, Loeschcke V (2006). Microsatellites provide insight into contrasting mating patterns in arribada vs. non arribada olive ridley sea turtle rookeries.. Molecular Ecology.

[61] Simmons LW, Beveridge M, Evans JP (2008). Molecular evidence for multiple paternity in a feral population of green swordtails.. Journal of Heredity.

