# Supplementary material for: Moderate Multiple Parentage and Low Genetic Variation Reduces the Potential for Genetic Incompatibility Avoidance Despite High Risk of Inbreeding
Source: PLoS One. 2012 Jan 3;7(1):e29636. doi: 10.1371/journal.pone.0029636 (PMC3250463; doi:10.1371/journal.pone.0029636)
Supplement: Table S1 — Skew analyses. PARENTAGE 1.0 estimates the contribution of each father to the offspring. For each iteration we calculated paternity skew as the sum of squared proportion of offspring assigned to each father (Simmons et al. 2007). For each number of fathers in the estimated range we tested the average skew against a simulated distribution. Simulations were done using the actual number of offspring in the given nest and equal probability for each father to sire the offspring. Each father was assigned 1 offspring before simulations to avoid fathers siring no offspring. (DOC) [file pone.0029636.s001.doc]

**Table S1. Skew analyses.**

|  | **# Fathers** | **1** | **2** | **3** | **4** | **5** |
| --- | --- | --- | --- | --- | --- | --- |
| **Nest** |  |  |  |  |  |  |
| **L-1 (13)** |  | 34 | 3277 | 1233 | 346 | 84 |
|  | estimated skew |  | 0,54 | 0,39 | 0,33 | 0,29 |
|  | expected skew |  | 0,53 | 0,37 | 0,29 | 0,24 |
|  | P-value |  | 0,23 | 0,37 | 0,09 | 0,06 |
| **L-2 (14)** |  | 4520 | 440 | 38 | 2 |  |
|  | estimated skew |  | 0,54 | 0,42 | 0,37 |  |
|  | expected skew |  | 0,53 | 0,37 | 0,29 |  |
|  | P-value |  | 0,39 | 0,1 | 0,02 |  |
| **L-3 (14)** |  | 372 | 851 | 2400 | 1013 | 267 |
|  | estimated skew |  | 0,55 | 0,38 | 0,32 | 0,29 |
|  | expected skew |  | 0,53 | 0,37 | 0,29 | 0,24 |
|  | P-value |  | 0,14 | 0,26 | 0,11 | 0,03 |
| **SB-1 (14)** |  | 0 | 4909 | 86 | 4 |  |
|  | estimated skew |  | 0,61 | 0,46 | 0,41 |  |
|  | expected skew |  | 0,53 | 0,37 | 0,29 |  |
|  | P-value |  | 0,04 | 0,03 | 0,01 |  |
| **SB-2 (11)** |  | 4998 | 2 |  |  |  |
|  | estimated skew |  |  |  |  |  |
|  | expected skew |  |  |  |  |  |
|  | P-value |  |  |  |  |  |
| **SB-3 (17)** |  | 1410 | 3331 | 236 | 20 | 3 |
|  | estimated skew |  | 0,52 | 0,38 | 0,34 | 0,28 |
|  | expected skew |  | 0,53 | 0,37 | 0,28 | 0,23 |
|  | P-value |  | 0,3 | 0,21 | 0,03 | 0,04 |
| **SB-4 (41)** |  | 3403 | 478 | 1096 | 23 |  |
|  | estimated skew |  | 0,55 | 0,38 | 0,27 |  |
|  | expected skew |  | 0,51 | 0,35 | 0,27 |  |
|  | P-value |  | 0,06 | 0,06 | 0,3 |  |
| **SB-5 (64)** |  | 0 | 3445 | 1535 | 20 |  |
|  | estimated skew |  | 0,53 | 0,35 | 0,27 |  |
|  | expected skew |  | 0,51 | 0,34 | 0,26 |  |
|  | P-value |  | 0,06 | 0,15 | 0,08 |  |
| **SB-6 (29)** |  | 355 | 4188 | 441 | 15 |  |
|  | estimated skew |  | 0,52 | 0,37 | 0,28 |  |
|  | expected skew |  | 0,52 | 0,35 | 0,27 |  |
|  | P-value |  | 0,26 | 0,23 | 0,28 |  |
| **SB-7 (33)** |  | 4998 | 2 |  |  |  |
|  | estimated skew |  |  |  |  |  |
|  | expected skew |  |  |  |  |  |
|  | P-value |  |  |  |  |  |
| **S1-1 (14)** |  | 4238 | 570 | 127 | 43 | 16 |
|  | estimated skew |  | 0,53 | 0,39 | 0,34 | 0,33 |
|  | expected skew |  | 0,53 | 0,37 | 0,29 | 0,24 |
|  | P-value |  | 0,39 | 0,21 | 0,05 | 0,01 |
| **S1-2 (34)** |  | 4211 | 751 | 31 | 6 |  |
|  | estimated skew |  | 0,52 | 0,36 | 0,28 |  |
|  | expected skew |  | 0,51 | 0,35 | 0,27 |  |
|  | P-value |  | 0,2 | 0,24 | 0,24 |  |
| **S1-3 (12)** |  | 4998 | 2 |  |  |  |
|  | estimated skew |  |  |  |  |  |
|  | expected skew |  |  |  |  |  |
|  | P-value |  |  |  |  |  |
| **S1-4 (60)** |  | 0 | 60 | 2591 | 2174 | 174 |
|  | estimated skew |  | 0,66 | 0,38 | 0,28 | 0,22 |
|  | expected skew |  | 0,51 | 0,34 | 0,26 | 0,21 |
|  | P-value |  | 0 | 0,01 | 0,03 | 0,08 |
| **S1-5 (15)** |  | 3994 | 750 | 195 | 45 | 11 |
|  | estimated skew |  | 0,54 | 0,38 | 0,30 | 0,29 |
|  | expected skew |  | 0,53 | 0,37 | 0,29 | 0,24 |
|  | P-value |  | 0,27 | 0,27 | 0,26 | 0,04 |
| **S1-6 (83)** |  | 4747 | 243 | 8 |  |  |
|  | estimated skew |  | 0,51 | 0,34 |  |  |
|  | expected skew |  | 0,51 | 0,34 |  |  |
|  | P-value |  | 0,37 | 0,38 |  |  |
| **S1-7 (34)** |  | 4441 | 517 | 37 | 5 |  |
|  | estimated skew |  | 0,52 | 0,36 | 0,26 |  |
|  | expected skew |  | 0,51 | 0,35 | 0,27 |  |
|  | P-value |  | 0,2 | 0,28 | 0,65 |  |
| **S1-8 (23)** |  | 1124 | 2799 | 807 | 207 | 42 |
|  | estimated skew |  | 0,53 | 0,37 | 0,29 | 0,25 |
|  | expected skew |  | 0,52 | 0,36 | 0,28 | 0,23 |
|  | P-value |  | 0,18 | 0,28 | 0,25 | 0,15 |
| **S1-9 (9)** |  | 1446 | 2188 | 884 | 307 | 118 |
|  | estimated skew |  | 0,56 | 0,41 | 0,35 | 0,31 |
|  | expected skew |  | 0,54 | 0,38 | 0,30 | 0,24 |
|  | P-value |  | 0,12 | 0,18 | 0,06 | 0,01 |
| **S1-10 (34)** |  | 4669 | 302 | 28 |  |  |
|  | estimated skew |  | 0,511383 | 0,356031 |  |  |
|  | expected skew |  | 0,513436 | 0,351266 |  |  |
|  | P-value |  | 0,37 | 0,28 |  |  |
| **S1-11 (10)** |  | 4466 | 450 | 66 | 15 |  |
|  | estimated skew |  | 0,57 | 0,44 | 0,40 |  |
|  | expected skew |  | 0,54 | 0,38 | 0,30 |  |
|  | P-value |  | 0,28 | 0,08 | 0,02 |  |
| **S1-12 (7)** |  | 1928 | 1574 | 831 | 391 | 186 |
|  | estimated skew |  | 0,57 | 0,43 | 0,37 | 0,33 |
|  | expected skew |  | 0,55 | 0,39 | 0,30 | 0,23 |
|  | P-value |  | 0,38 | 0,04 | 0,06 | 0 |
| **S2-1 (11)** |  | 4238 | 570 | 127 | 43 | 16 |
|  | estimated skew |  | 0,57 | 0,42 | 0,33 | 0,31 |
|  | expected skew |  | 0,54 | 0,38 | 0,29 | 0,24 |
|  | P-value |  | 0,18 | 0,11 | 0,08 | 0,01 |
| **S2-2 (28)** |  | 329 | 2760 | 1182 | 452 | 173 |
|  | estimated skew |  | 0,52 | 0,36 | 0,28 | 0,23 |
|  | expected skew |  | 0,52 | 0,36 | 0,27 | 0,22 |
|  | P-value |  | 0,33 | 0,38 | 0,33 | 0,38 |
| **S2-3 (20)** |  | 1361 | 1257 | 1266 | 671 | 299 |
|  | estimated skew |  | 0,54 | 0,37 | 0,30 | 0,24 |
|  | expected skew |  | 0,52 | 0,36 | 0,28 | 0,23 |
|  | P-value |  | 0,23 | 0,27 | 0,18 | 0,21 |
| **S2-4 (6)** |  | 4458 | 430 | 84 | 23 | 5 |
|  | estimated skew |  | 0,69 | 0,56 | 0,47 | 0,43 |
|  | expected skew |  | 0,56 | 0,39 | 0,30 | 0,23 |
|  | P-value |  | 0,13 | 0 | 0 | 0 |
| **S2-5 (9)** |  | 938 | 1813 | 1180 | 582 | 265 |
|  | estimated skew |  | 0,55 | 0,40 | 0,33 | 0,30 |
|  | expected skew |  | 0,54 | 0,38 | 0,30 | 0,24 |
|  | P-value |  | 0,45 | 0,38 | 0,06 | 0,01 |
| **S2-6 (28)** |  | 3217 | 861 | 602 | 229 | 60 |
|  | estimated skew |  | 0,53 | 0,36 | 0,28 | 0,23 |
|  | expected skew |  | 0,52 | 0,36 | 0,27 | 0,22 |
|  | P-value |  | 0,17 | 0,38 | 0,33 | 0,27 |
| **S2-7 (9)** |  | 3793 | 875 | 238 | 59 | 20 |
|  | estimated skew |  | 0,58 | 0,44 | 0,36 | 0,31 |
|  | expected skew |  | 0,54 | 0,38 | 0,30 | 0,24 |
|  | P-value |  | 0,12 | 0,06 | 0,06 | 0,01 |
| **S2-8 (15)** |  | 2481 | 2388 | 124 | 7 |  |
|  | estimated skew |  | 0,55 | 0,40 | 0,34 |  |
|  | expected skew |  | 0,53 | 0,37 | 0,29 |  |
|  | P-value |  | 0,27 | 0,12 | 0,05 |  |
| **S2-9 (12)** |  | 4886 | 114 |  |  |  |
|  | estimated skew |  | 0,61 |  |  |  |
|  | expected skew |  | 0,54 |  |  |  |
|  | P-value |  | 0,11 |  |  |  |
| **S2-10 (28)** |  | 4381 | 521 | 85 | 10 | 3 |
|  | estimated skew |  | 0,52 | 0,36 | 0,28 | 0,25 |
|  | expected skew |  | 0,52 | 0,36 | 0,27 | 0,22 |
|  | P-value |  | 0,33 | 0,26 | 0,19 | 0,07 |
| **S2-11 (8)** |  | 1723 | 1553 | 904 | 456 | 206 |
|  | estimated skew |  | 0,56 | 0,41 | 0,34 | 0,30 |
|  | expected skew |  | 0,55 | 0,39 | 0,30 | 0,24 |
|  | P-value |  | 0,22 | 0,14 | 0,2 | 0,04 |
| **S2-12 (35)** |  | 749 | 945 | 1879 | 1023 | 318 |
|  | estimated skew |  | 0,52 | 0,36 | 0,27 | 0,22 |
|  | expected skew |  | 0,51 | 0,35 | 0,27 | 0,22 |
|  | P-value |  | 0,17 | 0,29 | 0,31 | 0,31 |
| **S2-13 (23)** |  | 925 | 1401 | 1419 | 717 | 337 |
|  | estimated skew |  | 0,52 | 0,36 | 0,28 | 0,23 |
|  | expected skew |  | 0,52 | 0,36 | 0,28 | 0,23 |
|  | P-value |  | 0,38 | 0,33 | 0,3 | 0,36 |
| **S2-14 (27)** |  | 4110 | 688 | 163 | 33 | 6 |
|  | estimated skew |  | 0,52 | 0,36 | 0,28 | 0,23 |
|  | expected skew |  | 0,52 | 0,36 | 0,27 | 0,22 |
|  | P-value |  | 0,42 | 0,28 | 0,28 | 0,27 |
| **S2-15 (9)** |  | 1864 | 1662 | 875 | 356 | 146 |
|  | estimated skew |  | 0,56 | 0,41 | 0,34 | 0,30 |
|  | expected skew |  | 0,54 | 0,38 | 0,30 | 0,24 |
|  | P-value |  | 0,12 | 0,18 | 0,06 | 0,01 |
| **S3-1 (36)** |  | 1927 | 2081 | 785 | 175 | 26 |
|  | estimated skew |  | 0,52 | 0,35 | 0,27 | 0,23 |
|  | expected skew |  | 0,51 | 0,35 | 0,27 | 0,22 |
|  | P-value |  | 0,23 | 0,35 | 0,31 | 0,25 |
| **S3-2 (22)** |  | 2594 | 2170 | 204 | 31 |  |
|  | estimated skew |  | 0,52 | 0,37 | 0,32 |  |
|  | expected skew |  | 0,52 | 0,36 | 0,28 |  |
|  | P-value |  | 0,26 | 0,28 | 0,051 |  |
| **S3-3 (23)** |  | 755 | 2321 | 1278 | 445 | 131 |
|  | estimated skew |  | 0,53 | 0,36 | 0,28 | 0,24 |
|  | expected skew |  | 0,52 | 0,36 | 0,28 | 0,23 |
|  | P-value |  | 0,18 | 0,32 | 0,3 | 0,24 |
| **S3-4 (25)** |  | 4281 | 533 | 129 | 45 | 6 |
|  | estimated skew |  | 0,53 | 0,36 | 0,28 | 0,24 |
|  | expected skew |  | 0,52 | 0,36 | 0,27 | 0,23 |
|  | P-value |  | 0,22 | 0,28 | 0,28 | 0,13 |
| **S3-5 (27)** |  | 294 | 3107 | 1206 | 302 | 75 |
|  | estimated skew |  | 0,52 | 0,36 | 0,28 | 0,23 |
|  | expected skew |  | 0,52 | 0,36 | 0,27 | 0,22 |
|  | P-value |  | 0,22 | 0,35 | 0,28 | 0,24 |
| **S3-6 (19)** |  | 542 | 2528 | 1387 | 407 | 111 |
|  | estimated skew |  | 0,56 | 0,38 | 0,30 | 0,26 |
|  | expected skew |  | 0,52 | 0,36 | 0,28 | 0,23 |
|  | P-value |  | 0,15 | 0,21 | 0,15 | 0,09 |
| **S3-7 (19)** |  | 4706 | 279 | 15 |  |  |
|  | estimated skew |  | 0,52 | 0,36 |  |  |
|  | expected skew |  | 0,52 | 0,36 |  |  |
|  | P-value |  | 0,34 | 0,34 |  |  |
| **S4-1 (14)** |  | 4321 | 591 | 74 | 11 | 3 |
|  | estimated skew |  | 0,53 | 0,39 | 0,33 | 0,30 |
|  | expected skew |  | 0,53 | 0,37 | 0,29 | 0,24 |
|  | P-value |  | 0,39 | 0,26 | 0,11 | 0,02 |
| **S4-2 (39)** |  | 1453 | 3361 | 175 | 11 |  |
|  | estimated skew |  | 0,51 | 0,35 | 0,27 |  |
|  | expected skew |  | 0,51 | 0,35 | 0,27 |  |
|  | P-value |  | 0,32 | 0,38 | 0,4 |  |
| **S4-3 (15)** |  | 4298 | 642 | 52 | 8 |  |
|  | estimated skew |  | 0,54 | 0,40 | 0,31 |  |
|  | expected skew |  | 0,53 | 0,37 | 0,29 |  |
|  | P-value |  | 0,27 | 0,12 | 0,26 |  |
| **S4-4 (4)** |  | 2993 | 1217 | 544 | 246 |  |
|  | estimated skew |  | 0,66 |  |  |  |
|  | expected skew |  | 0,56 |  |  |  |
|  | P-value |  | 0 |  |  |  |
| **S4-5 (27)** |  | 4 | 4027 | 909 | 55 | 4 |
|  | estimated skew |  | 0,53 | 0,37 | 0,28 | 0,24 |
|  | expected skew |  | 0,52 | 0,36 | 0,27 | 0,22 |
|  | P-value |  | 0,22 | 0,23 | 0,21 | 0,14 |
| **S4-6 (4)** |  | 3346 | 1041 | 443 | 170 |  |
|  | estimated skew |  | 0,71 |  |  |  |
|  | expected skew |  | 0,56 |  |  |  |
|  | P-value |  | 0 |  |  |  |
